# A Novel “Endocrine Hormone”: The Diverse Role of Extracellular Vesicles in Multiorgan Insulin Resistance

**DOI:** 10.7150/ijms.97217

**Published:** 2024-08-06

**Authors:** Fangzhi Xu, Lin Dou, Dongni Yu, Xi Wu, Longteng Liu, Yong Man, Xiuqing Huang

**Affiliations:** 1 The Key Laboratory of Geriatrics, Beijing Institute of Geriatrics, Institute of Geriatric Medicine, Chinese Academy of Medical Sciences, Beijing Hospital/ National Center of Gerontology of National Health Commission, 100730, Beijing, P.R. China.; 2 Department of Dermatology, Beijing hospital, National Center of Gerontology; Institute of Geriatric Medicine, Chinese Academy of Medical Sciences, 100730, Beijing, P.R. China.

**Keywords:** extracellular vesicles, insulin resistance, intercellular communication, metabolic diseases, endocrine hormones, organokines

## Abstract

Insulin resistance is the primary contributor to the disruption in glucose homeostasis in the body, playing a significant causative role in many metabolic diseases. Insulin resistance is characterized by compensatory insulin secretion and reduced insulin responsiveness in target organs. Dysregulation of the interaction between insulin-secreting cells and insulin-responsive target organs is an important factor driving the progression of insulin resistance. Circulating endocrine hormones are important mediators mediating the interaction between insulin-secreting cells and insulin-responsive target organs. In addition to the classical hormones secreted by endocrine glands and organ-specific hormones secreted by metabolism-related organs (adipose tissue, muscle, liver, etc.), extracellular vesicles have been recognized as a novel class of endocrine hormones with a complex composition. Extracellular vesicles can transport signaling molecules, such as miRNAs and LncRNAs, to vital organs related to insulin resistance, in a manner akin to conventional hormones. The significant role in regulating the development of insulin resistance underscores the increasing interest in extracellular vesicles as essential contributors to this process. In this review, we summarize the three types of hormones (classical hormones, organokines and extracellular vesicles) that play a regulatory role in insulin resistance, and focus on the novel endocrine hormones, extracellular vesicles, to elaborate the mechanism of extracellular vesicles' regulation of insulin resistance progress from two aspects: the impact on insulin-secreting cells and the influence on insulin-responsive target organs. In addition, this paper outlines the clinical applications of extracellular vesicles in insulin resistance. A comprehensive understanding of the regulatory mechanisms and diagnostic status of the inter-organ network in insulin resistance has great potential to advance targeted therapeutic interventions and diagnostic markers, thereby benefiting both the prevention and treatment of insulin resistance.

## Introduction

Insulin resistance (IR) is an important cause of imbalance in the body's glucose homeostasis. It is prevalent in various metabolic diseases, and is considered to be the driving factor of metabolic syndrome 1, obesity, type 2 diabetes mellitus (T2DM) 2, cardiovascular disease 3, metabolic dysfunction related fatty liver disease (MAFLD) 4, polycystic ovary syndrome (PCOS) 5, Alzheimer's disease (AD) 6and some other tumors 7, all of which affect global public health 8.

IR, which is characterized by compensatory insulin secretion mediated by pancreatic β-cells and a decreased response of multiple insulin-responsive target organs, is a metabolic disease involving multiple organs including insulin-secreting organs and insulin-responsive target organs 7. Under physiological conditions, insulin-secreting cells and insulin-responsive target organs work together to coordinate interactions among insulin-related organs through metabolic regulatory signals, to maintain blood glucose levels in the body. Under pathological conditions, changes in the cargo and level of these regulatory signals occur, causing dysregulation of insulin-related organ interactions, which is an important factor driving the progression of IR 4.

Circulating endocrine hormones are significant classes that make up the metabolic regulatory network. These hormones include classical hormones secreted by specialized endocrine glands, such as insulin 9, growth hormone 10, and thyroxine 11; organokines secreted by important metabolic organs, such as adipokines, myokines, and hepatokines 12; and a novel endocrine hormone, which has recently received a great deal of attention ‒ extracellular vesicles (EVs) 13-15. Together, these hormone signals play a role in the interactions among organs related to IR 16.

EVs are vesicles secreted by a variety of cells that contain complex contents and are the new mediators that regulate organ-organ and cell‒cell interactions 17. Similar to classical hormones and organokines, EVs can transport specific cargos to target cells via blood or body fluids to perform physiological or pathological functions. Therefore, they are referred to as a new type of endocrine hormone 18-21.

This review focuses on the important role of EVs in disease pathogenesis, diagnosis and treatment, with a particular emphasis on EV-mediated interactions among vital organs related to insulin resistance.

## Insulin resistance is a metabolic disease involving multiple organs

IR refers to a disorderly biological response in which the insulin signaling pathway in target tissues is disrupted, resulting in a significant reduction in sensitivity to insulin 7. It is closely related to the dysregulation of insulin secretion and impaired insulin utilization. As a result, IR-related organs mainly include insulin-secreting organs and insulin-responsive target organs.

### Insulin-secreting organs/cells

The pancreas is an endocrine organ that plays a key role in the regulation of glucose homeostasis. Endocrine cells in the pancreas aggregate to form islets of Langerhans, which maintain glucose homeostasis by releasing a variety of hormones. The core hormone molecule insulin, which is secreted by pancreatic islet β-cells, is present throughout the pathological process of IR.

When IR occurs, the sensitivity of target tissues to insulin decreases, leading to a decrease in the efficiency of insulin in promoting glucose uptake and utilization, and an increase in body glucose levels. To maintain blood glucose homeostasis, pancreatic β-cells compensate for IR by either increasing the cell numbers or increasing the amount and/or frequency of insulin secretion. However, prolonged compensatory effects lead to a decrease in the number of pancreatic β-cells and/or gradual impairment of function 22.

### Insulin-responsive target organs

Insulin-responsive organs are distributed throughout the body. Among them, the liver, muscle, and adipose tissue are considered to be important target organs for IR. In addition, other organs such as the kidney, heart, ovary, and brain, also undergo IR and are involved in several pathophysiological processes through the target organ IR 2, 23.

After insulin is transported through the bloodstream to target organs, these organs respond to insulin's actions, contributing to the maintenance of the body's glucose homeostasis. Under normal physiological conditions, the precise regulation of target cell functions within target organs by insulin involves four sequential steps: insulin binding to its specific receptor, receptor substrate activation, signal transmission, and the generation of physiological effects 23.

When IR occurs, the insulin signaling pathway and its mediating effects on organs are disrupted. For example, in the case of liver IR, glycogen synthesis in the liver is reduced, gluconeogenesis is inhibited, and liver fat production is reduced. In skeletal muscle IR, glucose uptake by skeletal muscle decreases, and muscle glycogen synthesis is reduced. In adipose tissue IR, adipogenesis decreases and lipolysis increases. When these target organs exhibit reduced insulin sensitivity, they do not respond effectively to insulin, which is an important contributor to the sustained elevation of blood glucose level in the body 23.

Previous studies have explored the specific signaling pathway of IR in different target organs, which starts from the activation of insulin receptor in target cells to the final stages of glycolipid synthesis and decomposition. However, these studies are mostly focused on the specific role of a single target organ, and few addressing how cells/organs regulate and influence each other. In the early stage, the inter-organ communication was limited to classical hormone molecules. From the perspective of macro endocrine, the synergistic or antagonistic effects of different hormone molecules on insulin were discussed. 10, 11, 24-27. With the continuous updating of experimental methodology, researchers linked organokines and EVs with IR 13, 16. These molecules established connections between donor and recipient cells through "hormone" like effects, refining the way some signal molecules in the microenvironment communicate with different cells 28.

## Hormone mediators of cell/organ interactions related to insulin resistance

Intercellular/interorgan communication is the means by which an organism regulates its physiological functions and serves as a regulatory mechanism for many pathological processes. Mediators of cell/organ communication play a crucial role in the regulation of both physiological and pathological processes. In the physiological state, interorgan/intercell coordination and cooperation between cells are realized through signaling and interaction to maintain the homeostasis, development and functional performance of the organism. Dysregulated intercellular/organ communication is an important factor driving disease progression. Cells under stress or injury contribute to the disease process by releasing stress and danger signals that affect target cells, causing changes in the proliferation, metabolism or function of the cells involved 29, 30.

A complex network of interactions exists between insulin-secreting organs and insulin-responsive target organs, in which hormones play a major role. Classical hormones are chemical substances produced by specialized endocrine glands (e.g. pancreatic islets, adrenal glands, and thyroid glands) that are delivered via the bloodstream and initiate signaling either through cell surface receptors or through the cell membrane into the cell and bind to intracellular receptors to initiate signaling, thus playing a regulatory role in the organism 31. Classical hormonal mediators, involving insulin, glucagon 24, epinephrine 25, norepinephrine 26, cortisol 27, growth hormone 10 and thyroxine 11, play a central regulatory role in coordinating interactions between insulin-related organs and maintaining the body's blood glucose levels (**Table 1**).

With the deepening understanding of the endocrine system, the concept of hormones has expanded from a narrow definition of hormones secreted by endocrine glands to encompass a broader category of molecules or substances that exhibit characteristics similar to traditional hormones. This category includes organokines secreted by metabolism-related organs and a novel class of endocrine hormone secreted by various cells, known as EVs 13, 16.

Organokines, also known as organ hormones, refers to a complex array of cytokines secreted by organs such as adipose tissue, skeletal muscle, intestine and bone, which function as endocrine organs 12. Organokines share similarity with classical hormones in that they mediate interorgan crosstalk and participate in the regulation of organismal homeostasis through autocrine, paracrine, or endocrine actions 32-35. IR-related organ hormones primarily include Adipokines, Myokines, Hepatokines, Gut cytokines, and Osteokines 16, 36, 37, and certain non-tissue-specific inflammatory factors such as tumor necrosis factor (TNF)-α 38, interleukin (IL)-6^39^, and connexin (Cx)43 40 are also included. These factors are involved in IR processes by modulating insulin signaling pathways in insulin target organs (**Table 1**).

EVs are a collective term for lipid bilayer-enclosed particles released by cells 17, which are important mediators of cell/organ communication. There are several ways to classify EVs, and the most widely recognized classification in the academic community is to classify EVs into three categories based on their size, biogenesis, and synthesis process: exosomes, microparticles, and apoptotic vesicles. Exosomes are vesicular bodies with a diameter of 30-150 nm and a double-layer membrane structure, that are generated through the endocytosis-endosomal pathway and are released into the extracellular space by exocytosis. Microparticles, also known as microvesicles, which are 100-1000 nm in size, differ from exosomes in that they are formed by separation of cell membranes after direct outward budding. In addition, apoptotic bodies are membrane encapsulated vesicular bodies containing cytoplasm and organelles formed by cell shrinkage and fragmentation during the process of cell apoptosis, with a diameter of approximately 100-5000nm, which are released by the contraction of apoptotic cell membrane by budding 18. Due to their unique structure and formation mechanism, EVs can transport various contents, including proteins, nucleic acids and lipids, from donor cells to be released into the body fluids, which are ingested by recipient cells, so as to play the role of cell communication, information transmission and regulation of cell functions, and participate in a number of physiological and pathological processes 19. The uptake of EVs is divided into two steps. In the first step, EVs first connect with the receptor cell membrane through classical adhesion molecules (integrin, tetra transmembrane protein, lactoferrin, etc.) to bind to the surface receptor. In the second step, the receptor cells internalized EVs through macropinocytosis, phagocytosis, and clathrin/caveolin mediated endocytosis; or directly fuse with the cell membrane to release the contents into the target cells 41.

As a new type of endocrine hormone, EVs act in a classical endocrine hormone-like manner. They transmit signals through signaling molecules in the organism to regulate the function of target cells and maintain homeostasis 28. However, unlike classical hormones and organokines, which are typically single molecules, EVs carry complex cargos like proteins, nucleic acids, and lipids. This allows EVs to have a more detailed regulatory function in cell and organ interactions.^42^. In the physiological state, the cargos transported by EVs play a role in regulating the physiological functions of insulin-secreting organs and insulin-responsive target organs. However, in the pathological state, cells/organs influence the disease process by altering the composition of cargos in the EVs, either by decreasing the beneficial cargos or increasing the pathogenic cargos.

In the following sections, we will explore how EVs contribute to the regulation of disease progression and outline the clinical applications of EVs in IR-related diseases.

## The role of extracellular vesicles in the pathogenesis of insulin resistance

**Figure 1:**
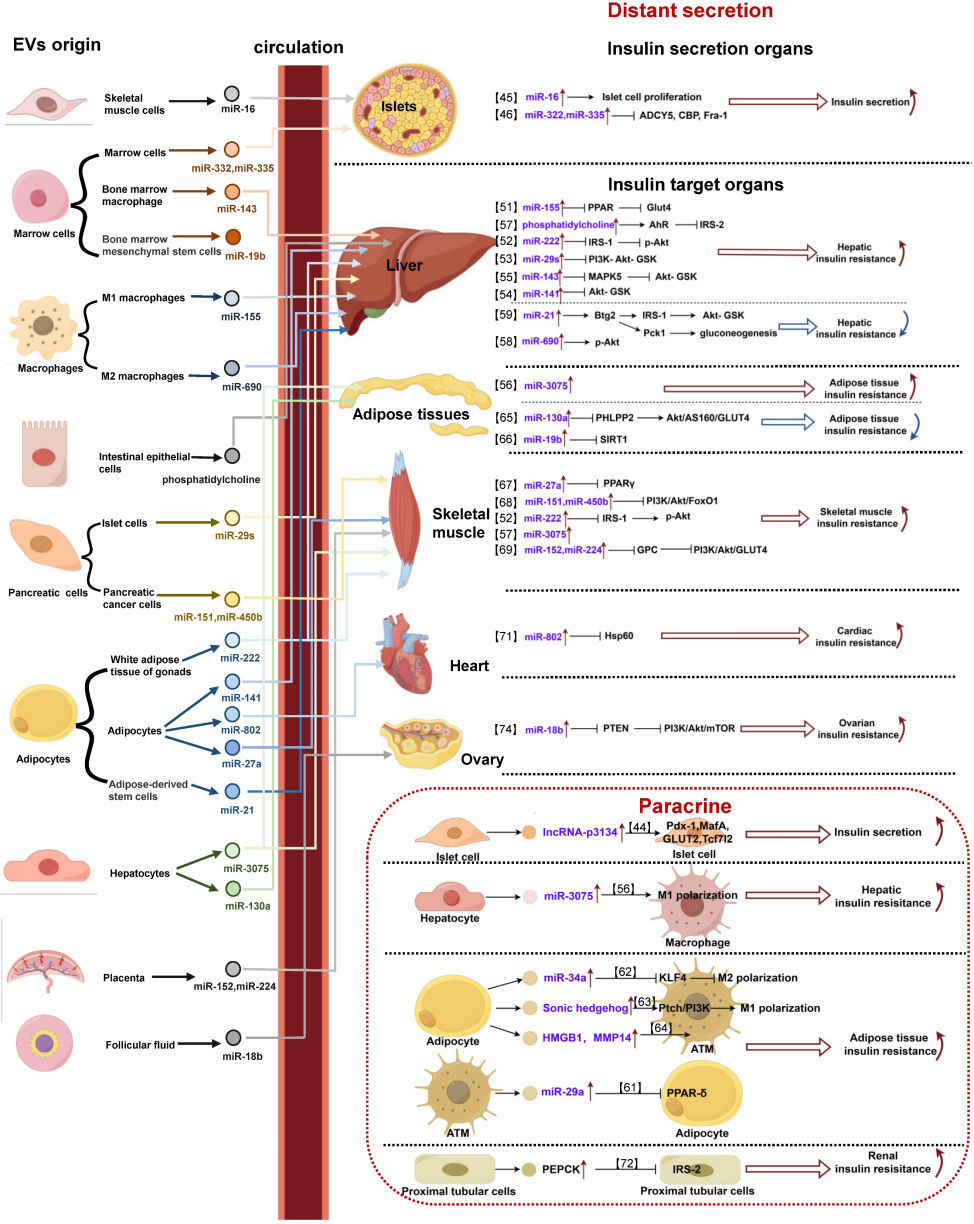
** EVs with specific cell source regulate IR in different organs by distant secretion or paracrine.** (1) Distant secretion of extracellular vesicles: extracellular vesicles with specific cargos secreted by different cells reach the target organ with blood circulation, and are taken up into the cell by the target cell, and then participate in the regulation of insulin secretion or insulin resistance of the target organ by acting on specific molecules. (2) Paracrine of extracellular vesicles: in the pancreas or insulin target organs (liver, adipose tissue, kidney), extracellular vesicles with specific cargos secreted by different cells act on adjacent cells through diffusion, and participate in regulating insulin secretion or insulin resistance of target organs.

In recent years, the regulatory role of EV-mediated cell/organ communication in IR has attracted much attention. As mentioned earlier, the pathological process of IR can be divided into two aspects: the compensatory secretion of insulin by pancreatic islet cells into the circulatory system, and the subsequent impact of insulin on various target organs, resulting in the biological effects of IR. In this section, we will delve into the regulatory mechanism of IR involving EVs, specifically exploring the contributions of EVs derived from different organ/tissue sources in the regulation of insulin secretion and their effects on insulin-responsive target organ (**Figure 1**).

### Extracellular vesicles are involved in the regulation of insulin secretion of pancreatic islets

In recent years, insulin secretion has been shown to be regulated not only by blood glucose levels and inflammatory factors, but also by EVs. EVs can target pancreatic islet cells via their cargos, directly affecting the secretion level of pancreatic islet cells or indirectly affecting the proliferation or apoptosis of pancreatic islet cells, thus participating in the regulation of insulin secretion 43.

In several animal models of IR-related diseases, researchers have found that EVs of well-defined sources enhance insulin secretion by transporting noncoding RNAs targeted to islet cells. For instance, In the T2DM mouse model, the content of lncRNA-p3134 in EVs released by mouse islet cells increased. These EVs act on adjacent islet β cells through the cell microenvironment, and promote compensatory insulin secretion by promoting the expression of PDX-1, MafA, GLUT2 and TCF7L2 44. In the high-fat fed (HFD) mouse model, EVs derived from skeletal muscle can enter the islet of Langerhans and enhance insulin secretion by carrying miR-16 to promote the proliferation of islet β cells 45. In the ovariectomized rat model simulating postmenopausal women, after treatment with liraglutide, the EVS derived from bone marrow are enriched with miR-322-3p and miR-335. These EVs enter the pancreas with blood and are internalized by islet cells to enhance insulin secretion by inhibiting target genes adcy5, CBP and Fra-1 46.

In contrast, some studies have identified circulating EVs of unknown origin in the circulatory system that are capable of triggering impaired insulin secretion. In obese mice, the expression of miR-26 in serum exosomes decreased. These circulating exosomes can target pancreatic islet cells and inhibit insulin secretion by inhibiting ten regulatory factors including mtpn, onecut2, cacna1c, plcb1, pja2, ESR1, EXT1, PAK2, GSK3 β and itga5 in pancreatic islet cells 47. However, in T2DM mice, the expression of miR-223 in serum exosomes is reduced, and it enters pancreatic islet cells with circulation, and insulin secretion is impaired by inhibiting FoxO1 and Sox6 pathways 48.

### Extracellular vesicles are involved in the regulation of insulin resistance in various insulin-responsive target organs

EVs carrying specific cargos to act on target cells are considered to be key factors in the development of IR in target organs. Currently, a large number of studies have focused on exploring the mechanisms by which EVs are involved in the regulation of IR in the three primary target organs-the liver, adipose tissue, and skeletal muscle. Whereas the mechanisms by which EVs are involved in IR in other target organs (e.g., heart, kidney, ovary, and brain) have not yet been elucidated 49, 50.

#### The role of extracellular vesicles in regulating hepatic insulin resistance

The liver, as an important site of venous return, receives an enriched supply of insulin through the portal vein, making the liver as one of the pivotal target organs affected by IR. When IR occurs in the liver, the inhibitory effect of insulin on hepatic glycogenolysis and glucose gluconeogenesis is weakened, which causes a rise in blood glucose.

EVs play a crucial role in the development of hepatic IR. Several studies have shown that EVs from various sources contribute to hepatic IR by delivering microRNAs (miRNAs) to hepatocytes. In the HFD mouse model, elevated free fatty acids (FFAs) increase miR-155 in the exosomes secreted by M1 macrophages in adipose tissue, and act on PPAR after being ingested by hepatocytes, thus blocking the expression of GLUT2, thereby aggravating hepatic IR 51. Elevated FFAs can also increase miR-222 in exosomes derived from white adipose tissue of mouse gonad, and then enter the liver, thereby down regulating the levels of IRS-1 and phosphorylated Akt in hepatocytes and promoting hepatic IR 52. Moreover, FFAs stimulation makes mouse islet cells secrete exosomes, carrying a significantly increased miR-29 family, targeting the liver with blood circulation, downregulating the phosphorylation of PI3K Akt GSK pathway in hepatocytes, thereby inducing hepatic IR 53.

In addition, in HFD mice, the expression of miR-141-3p in exosomes released from adipose tissue decreased significantly. When hepatocytes ingested these exosomes, their insulin sensitivity and glucose uptake ability decreased, promoting IR 54. However, in obese mice, exosomes derived from bone marrow macrophages carry elevated miR-143-5p into hepatocytes and induce liver IR by inhibiting the expression of mitogen activated protein kinase 5 (MPK5) 55; Liver parenchymal cells can secrete exosomes and carry elevated miR-3075, which acts on liver macrophages through the cell microenvironment, promotes macrophage infiltration and inflammatory reaction, thus inhibiting liver insulin sensitivity and participating in IR 56. In addition, EVs can also induce liver IR by delivering lipids to hepatocytes. In the HFD mouse model, exosomes derived from intestinal epithelial cells, containing phosphatidylcholine, were found to activate the hepatocyte AhR signaling pathway in an HFD mouse model. This activation downregulates IRS-2 expression and contributes to hepatic insulin signaling deficiency upon uptake by hepatocytes 57.

In contrast, several other studies have shown that EV-derived miRNAs can ameliorate hepatic IR once they enter hepatocytes. In HFD mouse model, the exocrine secretion secreted by M2 macrophages can act on hepatocytes with miR-690, upregulate Akt phosphorylation level and improve hepatic IR 58. In addition, in a PCOS rat model, exosomes derived from adipose stem cells carried miR-21-5p into hepatocytes, targeted the BTG antiproliferation factor 2 (Btg2), and induced the cAMP-response element binding protein (CREB) in hepatocytes to bind to the CREB cis element on the target gene promoter. This, in turn, increased the transcription of phosphoenolpyruvate carboxykinase (PEPCK) 1, promoting liver gluconeogenesis. Moreover, it activated the IRS1 and Akt pathways, thereby improving liver insulin sensitivity 59.

#### Extracellular vesicles are involved in the regulation of insulin resistance in adipose tissue

Adipose tissue serves as an important immune and endocrine organ, where inflammation and increased lipocalin secretion can lead to IR. When IR occurs in adipose tissue, fat synthesis decreases while catabolism is enhanced, leading to elevated blood lipids. Then, a large amount of free fatty acid is absorbed by the liver and converted to glucose, which subsequently leads to elevated blood glucose.

In recent years, several studies have reported that EVs, as a regulatory mediator, are also involved in the regulation of IR in adipose tissue 60. Among them, some studies have revealed that different kinds of cells in adipose tissue carried cargos, such as miRNA, via EVs to promote IR. In obese mice model, miR-29a was significantly elevated in exosomes secreted by adipose tissue macrophages (ATMs), which acted on adipocytes and induced adipose tissue IR by significantly inhibiting PPAR-δ 61. In addition, exosomes secreted by mature adipocytes promoted adipose tissue IR by translocating miR-34a into ATMs via paracrine secretion and inhibiting macrophage M2 polarization by suppressing the expression of Krüppel-like factor 4(KLF4) 62. As mentioned above, hepatocyte-derived exosomes carrying miR-3075 can also act on adipocytes via a compatible mechanism to inhibit adipose tissue insulin sensitivity to promote IR 56. In addition to miRNAs, EVs also carry DNA and proteins to induce adipose tissue IR. The exosomes secreted by adipocytes can carry the enhanced sonic hedgehog gene to target ATMs, and promote adipose tissue IR by mediating M1 macrophage polarization through the PTCH/PI3K signaling pathway 63. Adipocyte-derived exosomes carrying elevated high mobility group protein (HMG) B1 and matrix metalloproteinase (MMP) 14 acted on ATMs, leading to the induction of adipose tissue IR 64.

On the other hand, researchers have demonstrated that EVs derived from other tissues ameliorate adipose tissue IR by delivering miRNAs and targeting adipocytes. In HFD mouse model, exosomes secreted from hepatocytes could carry miR-130a-3p into adipocytes. The process subsequently inhibited the PH domain and leucine rich repeat protein phosphatase 2 (PHLPP2) gene, leading to the activation of the Akt/AS160/GLUT4 signaling pathway in adipocytes. This activation resulted in improved glucose tolerance and the subsequent restoration of adipose tissue IR 65. In addition, in an aging mouse model, miR-19b-3p released from mouse bone marrow mesenchymal stem cell-derived exosomes was taken up by adipocytes to ameliorate adipose tissue IR through the inhibition of silencing information regulatory factor 2-related enzyme 1(SIRT1) 66.

#### Extracellular vesicles are involved in the regulation of skeletal muscle insulin resistance

As the largest glucose storage and consumption organ in the body, skeletal muscle is the main target tissue for insulin. When IR occurs in skeletal muscle, the uptake and utilization of glucose by skeletal muscle cells are impaired, which in turn leads to elevated blood glucose. When IR occurs, the contractile and diastolic functions of skeletal muscle are impaired, which prevents the effective regulation of blood glucose levels and further leads to elevated blood glucose.

In various studies, researchers have independently reported that EVs secreted by different cells, carrying their specific miRNAs and targeting skeletal muscle, play a role in the promotion of its IR. In a T2DM mouse model, exosomes secreted by adipose tissue carrying elevated miR-27a into skeletal muscle cells induced skeletal muscle IR by inhibiting the target gene PPAR-γ 67. In a mouse model of pancreatic cancer, increasing exosomal miR-151-3p and miR-450b-3p derived from pancreatic cancer cell were absorbed by C2C12 cells in skeletal muscle myotubes. These miRNAs promoted skeletal muscle IR by inhibiting PI3K/Akt/FoxO1 signaling pathway 68. As mentioned above, exosomes from gonadal white adipose tissue originating from HFD-fed mice carry miR-222 into skeletal muscle cells, where it induces skeletal muscle IR by a similar molecular mechanism 52. Hepatocyte-derived exosomal miR-3075 can also promote skeletal muscle IR 56. In human studies, placenta-derived exosomes from patients with diabetic pregnancies were found to transport miR-152-3p and miR-224-5p into human skeletal muscle cells. These miRNAs targeted the regulation of glypican (GPC) genes, resulting in the inhibition of the insulin signaling PI3K/Akt/GLUT4 pathway and a reduction in skeletal muscle glucose uptake, thereby inducing IR 69.

In addition, some unknown EVs carrying miRNAs were also found in the circulatory system to participate in the induction of skeletal muscle IR. For instance, the circulating exosomes of T2DM patients highly express miR-20b-5p. After entering human skeletal muscle cells, these exosomes downregulate the activity of glycogen synthase by inhibiting the expression of Akt interacting proteins and signal transduction and transcription activators, and subsequently participate in the occurrence of skeletal muscle IR 70. However, how EVs ameliorate skeletal muscle IR remains to be further investigated.

#### Extracellular vesicles are involved in the regulation of insulin resistance in other target organs

##### Extracellular vesicles are involved in the regulation of cardiac insulin resistance

Insulin regulates the uptake and utilization of glucose by cardiomyocytes, as well as the recycling of calcium ions by the sarcoplasmic reticulum of cardiomyocytes. When IR occurs in the heart, the uptake and utilization of glucose by the heart are reduced, leading to elevated blood glucose and impaired diastolic and systolic function. However, there is only one study on the mechanism which EVs are involved in regulating cardiac IR. In this article, Wen et al. found in a neonatal rat model that hypertrophic adipocyte-derived exosomes highly expressed miR-802-5p, and that these exosomes entered ventricular myocytes and targeted silencing of heat shock protein (HSP) 60, thereby inducing cardiomyocyte IR and attenuating the insulin-sensitizing effects of lipocalin 71.

##### Extracellular vesicles are involved in the regulation of renal insulin resistance

The kidney is an important component in the regulation of glucose metabolism, and insulin promotes renal glucose uptake and utilization. When IR occurs in the kidney, in addition to impaired renal glucose utilization, renal function significantly changes, and even progresses to renal insufficiency. Up to date, no study has clearly reported the role that EVs play in the mechanism of renal IR, but some studies have shown that exosomes secreted by human proximal tubule cells, which carry large amounts of PEPCK, enter surrounding proximal tubule cells, inhibit IRS-2, and impair renal gluconeogenesis 72.

##### Extracellular vesicles are involved in the regulation of ovarian insulin resistance

Insulin is involved mainly in glucose metabolism and hormone synthesis in the ovary. When IR occurs, in addition to the decrease in glucose uptake and utilization by ovarian cells, hyperinsulinemia also promotes the production of excessive androgens in the adrenal gland and ovary, and leads to a decrease in sex hormone globulin levels and an increase in free testosterone by inhibiting liver synthesis, thus contributing to the occurrence of PCOS 73.

Due to the lack of relevant studies, the role of EVs in ovarian IR needs to be further clarified. In PCOS rats, miR-18b-5p is enriched in exosomes in follicular fluid. After these exosomes are ingested by ovarian granulosa cells, they target PTEN and PI3K/Akt/mTOR signaling pathway mediated by PTEN, thereby inhibiting ovarian IR 74. However, there is an unknown source of exosomes in the plasma of PCOS mice. The expression of miR-126-3p in these exosomes is increased and acts on ovarian granulosa cells to participate in ovarian IR by inhibiting platelet-derived growth factor receptor β (PDGFR β) and its downstream PI3K/Akt pathway 75.

##### Extracellular vesicles are involved in the regulation of insulin resistance in the brain

Insulin regulates cerebral vascular function and is also involved in maintaining brain proteostasis, influencing amyloid β-peptide clearance and tau protein phosphorylation. When brain IR occurs, brain's bioenergetics are severely affected. Specifically, when brain tissues have difficulty utilizing glucose, the brain adopts an energy-saving mechanism to survive by inhibiting functions such as learning and memory, which can cause cerebral atrophy or long-term AD 76. In addition, brain IR is involved in the onset and development of Parkinson's disease by causing mitochondrial dysfunction, oxidative stress, and neuroinflammation, and is closely associated with cognitive dysfunction in Parkinson's disease 77. However, no study has reported the specific role of EVs in cerebral IR until now, but some clinical studies have shown that neurogenic exosomes can be detected in the blood, which suggests that EVs can be involved in the pathological process of IR in the brain 78.

In conclusion, EVs from different cell sources act on different target cells in a similar "hormone like" manner. Under the pathological condition of IR, high glucose, high fat and other stimulating factors act on the donor cells to change the expression levels of EVs contents. These EVs reach the specific recipient cells through remote secretion of blood, paracrine secretion of tissue fluid or microenvironment autocrine secretion, and are taken up by the recipient cells. Once inside, they release the effective content in the recipient cells to participate in the regulation of IR. However, because EVs carry different effectors, their mechanisms of action in target cells are completely different. For example, non-coding RNAs participate in IR signal pathway by inhibiting downstream target genes, while proteins or lipids directly interact with signal molecules to affect IR signal pathway. It is worth noting that current determinations of effector content are mostly based on detecting expression differences in donor cells, recipient cells and EVs. These contents are highly correlated with donor cells and participate in the regulation of the proliferation, secretion and other functions of donor cells. However, whether these effector content determines the targeting of EVs to some extent is worth further exploration. In addition, the interaction between adhesion molecules on EVs and specific receptors on the target cytoplasmic membrane makes the secretion of EVs have a certain targeting. However, due to the limitations of experimental technology, current research on the targeted uptake of EVs by recipient cells is limited, and clear conclusions are still lacking.

## The role of extracellular vesicles in the diagnosis and treatment of insulin resistance

### Diagnostic potential of extracellular vesicles in insulin resistance related diseases

**Figure 2:**
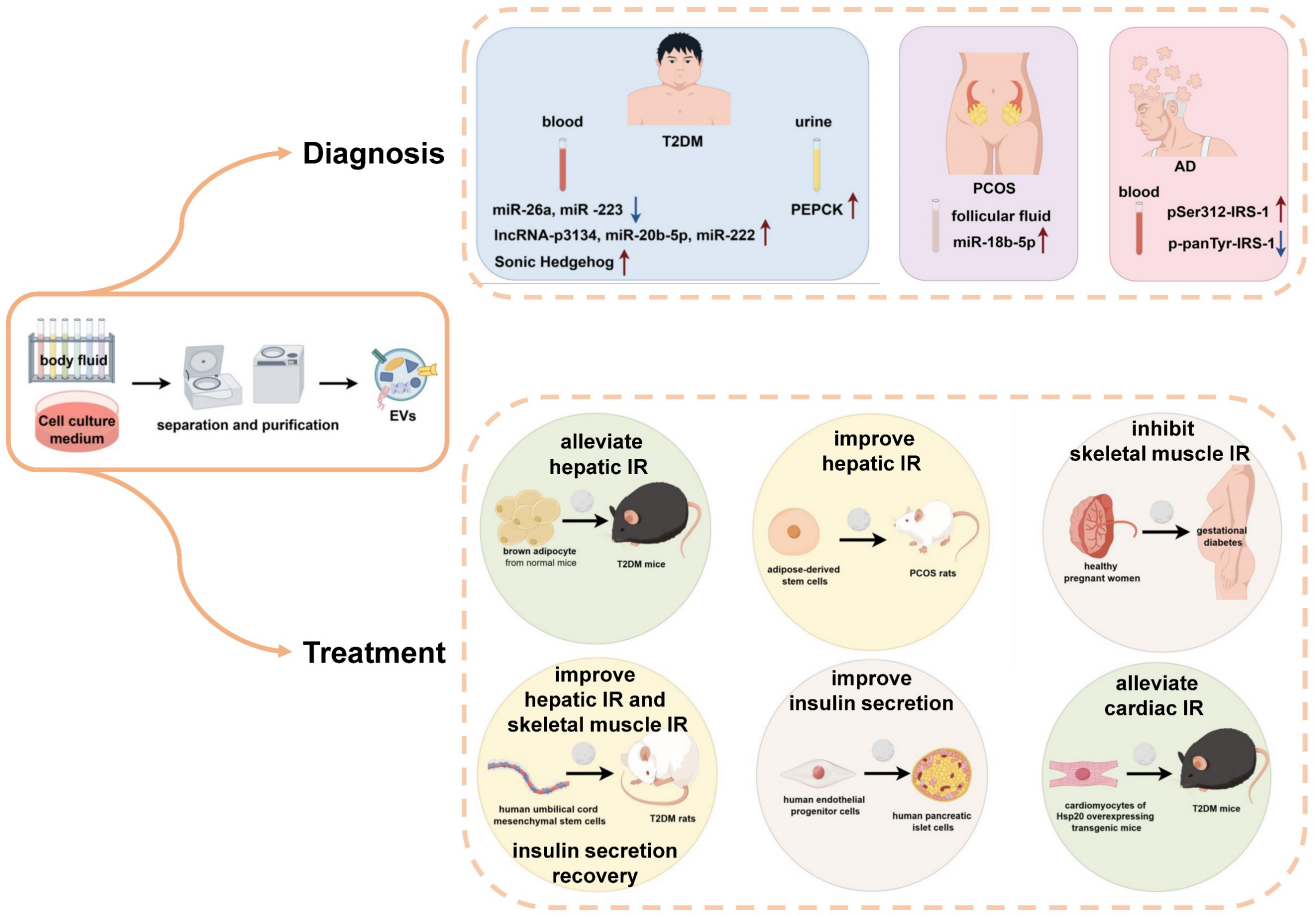
** The application of EVs in the diagnosis and treatment of IR related diseases.** The application of extracellular vesicles in insulin resistance related diseases is mainly divided into two types: as a biomarker for diagnosis and participation in treatment. Starting from the diagnostic biomarkers, the extracellular vesicles are isolated and purified from body fluid, and the content of specific cargos was detected. The increase or decrease of these cargos suggested the related progress of type 2 diabetes, polycystic ovary syndrome and Alzheimer's disease. From the perspective of treatment, after the isolation and purification of extracellular vesicles in body fluid or cell culture medium, using these extracellular vesicles to treat patients or animals with insulin resistance related diseases can significantly relieve the symptoms of insulin resistance in their insulin target organs (liver, skeletal muscle and heart) or improve insulin secretion.

EVs are widely present in various biological fluids, including blood and urine, and the contents of these vesicles are closely correlated with disease states. Due to their unique structure, EVs exhibit remarkable stability in body fluids, enduring long-term storage, repeated freeze‒thaw cycles, and extreme pH conditions. Therefore, EVs are expected to play an important role as non-invasive markers for the diagnosis and early screening of IR in the body (**Figure 2**).

Serum EVs play an important role in the diagnosis of T2DM. Several studies have reported that the serum exosomal miR-26a is significantly lower in overweight individuals than in healthy individuals and negatively correlates with the clinical features of T2DM 47. Additionally, miR-223 expression in the serum EVs is reduced in T2DM patients compared to healthy individuals 48. Conversely, levels of lncRNA-p3134, miR-20b-5p, and miR-222 were elevated in the serum EVs of patients with T2DM 44, 54, 70, and the population of sonic hedgehog positive exosomes in the serum of T2DM patients increased significantly 63. Urinary exosomes are also valuable in the diagnosis of pre-T2DM, where PEPCK is significantly elevated and positively correlates with systemic insulin sensitivity 72.

In terms of PCOS diagnosis, miR-18b-5p levels are elevated in follicular fluid exosomes from female patients 74. In terms of AD diagnosis, AD patients have higher expression of pSer_312_-IRS-1 and lower expression of p-panTyr-IRS-1 in peripheral blood exosomes, and brain volume in AD patients is positively correlated with P-panTyr-IRS-1 and negatively correlated with pSer_312_-IRS-1^77^, ^78^.

The mechanism by which these EV-derived small molecules target individual cells and participate in the IR of each target organ has been described in detail in the previous section, and will not be repeated here. However, their sensitivity and specificity need to be further verified in clinical tests, but provide a new direction for the diagnosis and screening of IR-related diseases.

### Therapeutic perspectives of extracellular vesicles in insulin resistance-related diseases

With the deepening of our understanding of EVs, EVs have the following advantages in disease therapy. Firstly, EVs possess tough lipid bilayer vesicles to maintain bioactivity while being repeatedly manipulated. Secondly, they are immune-tolerant, easy to manipulate, and highly permeable to biological barriers. Therefore, EVs hold great promise for the treatment of a wide range of diseases involving IR (**Figure 2**).

In clinical therapeutic applications, one study revealed that using EVs secreted by human endothelial progenitor cells to treat human pancreatic islet cells *in vitro* can improve glucose stimulated insulin secretion and reduce the β apoptosis rate 79. Another study reported the inhibition of skeletal muscle IR in patients with diabetic gestation by administering placental exosomes secreted from the body fluids of healthy pregnant women 69.

On the other hand, in animal studies, injection of brown adipocyte-derived exosomes from normal mice into T2DM mice was found to alleviate hepatic IR and impaired glucose tolerance 80. After injecting EVs derived from human umbilical cord mesenchymal stem cells into T2DM rats, the phosphorylation of IRS-1 and Akt in skeletal muscle and liver was restored, which promoted the expression and membrane translocation of GLUT4 in muscle, and the synthesis of glycogen in the liver. Moreover, these exosomes could inhibit β-cell apoptosis and promote the recovery of insulin secretion function 81-83. Exosomes secreted by cardiomyocytes from Hsp20 overexpressing transgenic mice were collected and injected into mice with diabetic cardiomyopathy. These exosomes carry a large number of phosphorylated Akt molecules, which can alleviate IR in cardiomyocytes 84. The application of exosomes derived from adipose derived stem cells in the treatment of rats with PCOS can improve liver glucose metabolism in rats, thereby alleviating the symptoms of PCOS, through a therapeutic mechanism as described previously 59. Although, there are few reports on the use of EVs for the treatment of IR-associated diseases, they also provide new ideas for future disease treatment.

## Conclusion

As our understanding of EVs deepens, it becomes increasingly clear that EVs carry specific small molecules and play important roles in the development and progression of IR in various organs of the body, essentially acting as a novel endocrine hormone in intercellular communication. Although research on the use of EVs in the diagnosis and treatment of IR-related diseases is still in the early stage, their great potential should not be underestimated. Therefore, further investigation into the biological source, release, targeted regulation of EVs, their underlying regulatory mechanisms, and the exploration of new molecular markers associated with EVs in the diagnosis and treatment of IR-related diseases will undoubtedly have far-reaching significance for the prevention and treatment of IR-related diseases.

## Figures and Tables

**Table 1:** The role of classical hormones and organokines in insulin resistance

Classification	Scientific Name		Molecular Mechanism	Effect in insulin resistance (+ : promote; - : inhibit)
**Classical hormone**	Glucagon		Promote fat decomposition and oxidation; Promote insulin secretion in the body.	**+**
	Epinephrine, Norepinephrine		Bind to insulin receptor and inhibit the biological activity of insulin; Promote fat decomposition and increase the level of free fatty acids, thus inhibiting insulin stimulated glucose utilization.	**+**
	Cortisol		Inhibit insulin secretion in pancreas β cells; Promote gluconeogenesis in liver and muscle; Promote fat decomposition; Promote the decomposition of muscle protein, increase the level of amino acids, thereby stimulating insulin secretion.	**+**
	Growth hormone		Promote the uptake and utilization of glucose in muscle and adipose tissue; Inhibit insulin secretion in pancreas β cells.	**-**
			Promote the decomposition of adipose tissue; Promote gluconeogenesis in liver and muscle.	**+**
	Thyroxine		Promote protein synthesis; Increase glucose utilization in muscle and adipose tissue; Inhibit insulin secretion in pancreas β cells; Promote the decomposition of adipose tissue.	**-**
			Promote protein metabolism; Promote gluconeogenesis in liver and muscle.	**+**
**Organokines**	Adipokines	Leptin	Enhance glucose uptake in brown adipose tissue and skeletal muscle.	**-**
			Inhibit hepatic glucose output; Inhibit the secretion of corticosterone and glucagon.	**+**
		Adiponectin	Improve inflammatory response and oxidative stress; Enhance the utilization of glucose and fatty acids in skeletal muscle; Inhibit gluconeogenesis and glycogen decomposition in the liver.	**-**
		ZAG	Restore the damaged hepatic IRS/Akt signal transduction induced by HFD or palmitic acid.	**-**
		CCN4	Weaken the effect of insulin on the phosphorylation of insulin receptor B, Akt and GSK3β	**+**
	Myokines	Irisin	Improve the lipid and glucose metabolism of skeletal muscle and liver through PI3K/Akt and AMPK pathways; Increase β Cell viability through Akt/Bcl2 signaling pathway.	**-**
		MSTN	Degrade IRS1 protein by CBLB in a Smad3 dependent manner.	**+**
		BABA	Reduce gluconeogenesis through IRS1/Akt and AMPK pathways.	**-**
	Hepatokines	Fetuin-A	Increase the production of non-esterified fatty acids, inflammatory precursors of IL-6, IL1β and TNF-α in macrophages and adipocytes, and decrease the production of anti-inflammatory cytokines such as lipocalin; Inhibit the tyrosine kinase of insulin receptor and destroy the downstream phosphorylation pathway of insulin receptor; As an endogenous ligand of Toll like receptor 4, saturated fatty acids induce pro-inflammatory signals and IR through this ligand.	**+**
		FGF21	Inhibit SREBP1c-mediated lipogenesis and hepatic gluconeogenesis through AMPK - SIRT1 pathway.	**-**
		Seleno protein P	unclear	**-**
	Gut cytokines	FGF15/19	Stimulate glycogen synthesis.	**-**
			Reduce glycogen production by inactivating GSK3 and CREB	**+**
		GLP-1	Enhance insulin sensitivity through PKC/PPARγ signaling.	
	Osteokines	Osteopontin	Inhibit STAT3 and increase expression of the PEPCK and glucose-6-phosphatase.	**+**
		Osteocalcin	Inhibit NF-KB signaling pathway to attenuate endoplasmic reticulum stress.	**-**
	inflammatory cytokines	TNF-α	Weaken insulin signaling through phosphorylation of serine to induces IR in adipocytes and peripheral tissues.	**+**
		IL-6	Mediate GLUT4 translocation and fatty acid oxidation via the AMPK pathway in muscle and fat; Increase hepatic glucose output through increased hepatic glycogenolysis, gluconeogenesis, and glucose release; Stimulate insulin secretion in β-cells by increasing glucagon-like peptide-1 in intestinal L-cells.	**-**
		Cx43	mediate an increase in intercellular coupling to synergize endoplasmic reticulum stress to promote hepatocyte IR.	**+**

## Abbreviations

AD: Alzheimer's disease
Adcy5: adenylate cyclase 5
Akt/PKB: protein kinase B
ATMs: adipose tissue macrophages
Bcl2: B-cell lymphoma-2
Bhlhe22: basic helix-loop-helix family, member e22
Btg2: anti-proliferation factor 2
Cacna1c: calcium channel, voltage-dependent, L type, alpha 1C subunit
CBLB: biquitin-protein ligase B
CBP: sarcoplasmic calcium-binding protein
CREB: cAMP-response element binding protein
Crem: cAMP responsive element modulator
Cx: connexin
Esr1: estrogen receptor 1
EVs: extracellular vesicles
Ext1: exostosin glycosyltransferase 1
FFAs: free fatty acids
FOXO1: forkhead box O1
FRA1: P-loop containing nucleoside triphosphate hydrolases superfamily protein
GLUT: glucose transporter protein
GPC: glypican
GSK: glycogen synthase kinase
GSV: glucose transporter protein type 4 storage vesicles
Hes1: hes family bHLH transcription factor 1
HFD: high-fat-diet
HMG: high mobility group protein
HSP: heat shock protein
IL: interleukin
Insm1: INSM transcriptional repressor 1
Insm2: INSM transcriptional repressor 2
IR: insulin resistance
IRS: insulin receptor substrate
Itga5: integrin alpha 5 (fibronectin receptor alpha)
KLF4: Krüppel-like factor 4
MafA: MAF bZIP transcription factor A
MAFLD: metabolic dysfunction related fatty liver disease
miRNA: microRNA
MMP: matrix metalloproteinase
MPK5: mitogen-activated protein kinase 5
mTORC2: mechanistic target of rapamycin complex 2
Mtpn: myotrophin
Neuro D: neurogenic differentiation Factor 1
NF-KB: nuclear factor-k-gene binding
Nkx2-2: NK2 homeobox 2
Nkx6.1: NK6 homeobox 1
Onecut2: one cut domain, family member 2
Pak2: p21 (RAC1) activated kinase 2
Pax6: paired box 6
PCOS: polycystic ovary syndrome
PDE3B: phosphodiesterase 3B
PDGFRβ: growth factor receptor β
PDK1: phosphatidylinositol-3-phosphate-dependent kinase-1
Pdx1: pancreatic and duodenal homeobox 1
PEPCK: phosphoenolpyruvate Carboxykinase
PHLPP2: PH domain and leucine rich repeat protein phosphatase 2
PI3K: phosphatidylinositol-3-OH kinase
PIP2: phosphatidylinositol-4,5-bisphosphate
PIP3: phosphatidylinositol-3,4,5-trisphosphate
Pja2: praja ring finger ubiquitin ligase 2
Plcb1: phospholipase C, beta 1
PP: protein phosphatase
p-panTyr-IRS-1: Phospho- Pan-Tyrrosine-Insulin Receptor Substrate 1
PPAR: peroxisome proliferator-activated receptor
p-Ser_312_-IRS-1: Phospho- Serine_312_-Insulin Receptor Substrate 1
Pten: phosphatase and tensin homolog
SIRT1: silencing information regulatory factor 2-related enzyme 1
Smad3: SMAD family member 3
Sox6 SRY: (sex determining region Y)-box 6
SREBP-1c: sterol regulatory element binding protein 1c
STAT3: signal transducer and activator of transcription 3
Stx3: syntaxin 3
T2DM: type 2 diabetes mellitus
Tcf7l2: transcription factor 7 like 2
TNF: tumor necrosis factor
